# Dual-stage ultrasound application for rice bran protein extraction: A novel method to improve yield, functional properties, and nutritional profile

**DOI:** 10.1016/j.ultsonch.2025.107681

**Published:** 2025-11-14

**Authors:** Saydul Md Safwa, Nikitha Modupalli, Md Mahfuzur Rahman

**Affiliations:** Department of Food Science, University of Arkansas, 2650 N. Young Ave., Fayetteville, AR 72704, United States

**Keywords:** Rice bran protein, Ultrasound-assisted precipitation, Protein solubility, Functional properties, Nutritional profile

## Abstract

While ultrasound-assisted protein extraction is well-studied, the application of ultrasound in both the extraction and precipitation stages has not been explored. This study explored the ultrasound (U)-assisted alkaline extraction (AE) at pH 9.5 and ultrasound-assisted isoelectric precipitation (UIP) at pH 3.5 on the rice bran protein yield, functionality, and nutritional quality. The UAE and conventional precipitation (UAE-IP) yielded the highest protein yield of 65.7 %, while conventional extraction yielded 38.9 %. FTIR showed that UAE-IP increased β-sheets by 96.8 % while AE-UIP decreased the α-helices by 42.4 %. Although UAE-IP showed no significant change in surface hydrophobicity, UAE-UIP decreased it by 34.7 % to the control. Consequently, functional properties such as solubility, emulsifying, and foaming capacities were significantly improved by 5 %, 0.05, and 3 % respectively. AE-UIP exhibited the highest solubility at pH 7, while UAE-IP showed better solubility at acidic and alkaline pHs. UAE-UIP demonstrated superior emulsion and foaming properties. Ultrasound application enhanced protein digestibility, with AE-UIP showing the highest digestibility of 96.91 %. However, dual-stage ultrasound reduced protein digestibility and subsequently affected the amino acid score (AAS). The amino acid profile varied with extraction methods, with AE-IP showing the highest levels of essential amino acids. AE-IP and UAE-IP showed no significant difference in PDCAAS (protein digestibility–corrected amino acid score) and AAS, whereas AE-UIP and UAE-UIP reduced them to 0.9 and 0.6, respectively. In summary, while ultrasound-assisted extraction enhances protein yield and certain properties, ultrasound-assisted precipitation shows great promise in further enhancing mass yield and functionality, which underscores the importance of a dual-stage ultrasound.

## Highlights

• Ultrasound during extraction showed the highest rice bran protein yield of 65.7 %.
• Multi-stage ultrasound applications significantly increased β-sheets and decreased α-helices.
• Ultrasound-assisted methods significantly improved solubility, emulsifying, and foaming properties.
• Ultrasound during precipitation showed the highest digestibility at 96.91 %.
• Multi-stage ultrasound applications showed a promise to improve protein yield and quality.

## Graphical abstract

**Figure:**
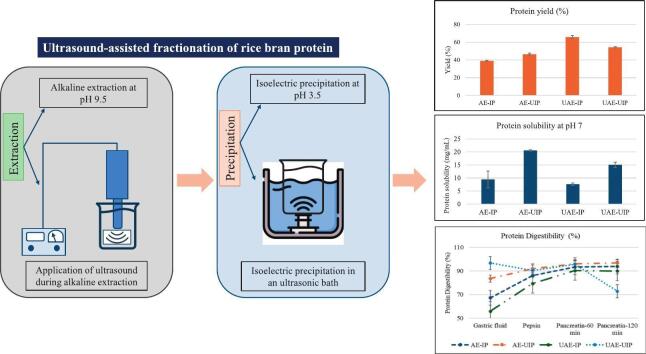

## 1 Introduction

The plant-based protein industry has increasingly focused on pulse proteins, including peas, chickpeas, lentils, and beans. However, pulses are low in sulfur-containing amino acids; cereal proteins are typically higher in sulfur-containing methionine and cysteine. In addition, cereal proteins from rice and wheat exhibit better functional properties, such as gelling and foaming, which can be advantageous in certain foods [1]. Cereal proteins are known to have lower allergenicity and better bioavailability [2]. Among cereal protein sources, wheat protein or gluten is dominating the plant-protein market; however, rice protein is significantly underutilized.

Rice bran, a by-product of rice milling, contains approximately 15 % protein, 15 % fiber, and 50 % carbohydrate [3]. The USA produces approximately 20.3 million metric tons (MMT) of rice bran annually [4], most of which is sold for around 10 cents per pound for use in pet food. This valuable resource is underutilized as a source of plant-based ingredients. A primary contributing factor to this underutilization is the complexity and inefficiency of existing protein extraction processes. The conventional rice bran protein (RBP) extraction method includes two stages: (1) alkaline extraction at pH 9.0 by solubilizing protein, which addresses protein yield, followed by (2) isoelectric precipitation (IEP) at pH 4.5 by coagulating protein, which addresses protein purity. This method often produces low protein yield and reduced purity: 13.8 % yield and 45–47 % purity [5]; 13.2 % yield and 37.6 % purity [6]; 24.02 % protein recovery [7]; and 39.4–44.0 % purity [8]. Furthermore, the high fiber content in rice bran dissolves in alkaline solutions, forms protein-fiber complexes during the precipitation, and reduces protein purity [9]. Enzymatic extraction of RBP offers potential advantages over conventional methods, but it also faces several challenges. Enzymatic extraction often results in extensive hydrolysis, forming small peptides that may alter the functional properties, thereby, product development performance [10]. Additionally, the process can be costly due to operational costs and the need for specific conditions. Optimizing enzyme type, concentration, and reaction parameters to balance yield and functionality remains an ongoing challenge for researchers [11,12].

Apart from enzymatic extraction, novel processing techniques such as high hydrostatic pressure (HHP) and microwave-assisted extraction were employed to enhance the extraction process. HHP pretreatment is an emerging technique that improves extraction, modifies protein by breaking down complex aggregates, and enhances functionality. Applying 200 MPa pressure during extraction has improved the protein solubility from 22.5 % to 43.7 % at pH 7.0. Further, gelation improved, but only at higher concentrations, and foaming properties were decreased at a pressure 600 MPa [13].

Among novel methods, ultrasound-assisted extraction (UAE) has emerged as an effective technique for improving RBP extraction. Ultrasound generates acoustic streaming within liquids, resulting in cavitation during the rapid formation and collapse of bubbles. This process produces high shear and mechanical energy, thus, disrupting the complex of the starch-protein-fiber matrix [14]. In addition, it will disturb the hydrophilic layers surrounding the proteins and makes them prone to aggregation during precipitation [15]. Ultrasound-assisted alkaline (pH 11.0) extraction has been studied and found to hold promise for increasing the yield and functional properties of RBP [16]. For instance, UAE resulted in a 22.04 % higher RBP yield and 6.19 % higher protein in the extracted material compared to alkaline extraction, while being 30 times faster [15]. Additionally, the UAE achieved protein yields of up to 64.5 % from rice bran, with the initial extraction rate and extraction constant being 3.48 and 2.20 times higher, respectively, than those of the conventional method [17]. In addition, UAE of soy proteins showed an increase in yield from 71 % to 81 % [18]. The same study also reported decreased protein solubility at pH 7.0 and reduced emulsification capacity after sonication, which was associated with increased exposure to hydrophobic groups. Similarly, ultrasound-assisted extraction has been reported to reduce solubility, emulsifying activity, and foaming stability in both soy [19] and rice bran protein [17]. In the studies mentioned above, ultrasound has been applied as a pretreatment to alkaline extraction to disrupt the cell membrane and enhance protein release into the extracting solvent. However, problems remain in precipitating all dissolved protein from the extracting solvent. This study hypothesizes that ultrasound-assisted extraction can disrupt rice bran cell walls and improve protein release in the extracting solvent, and ultrasound-assisted precipitation can facilitate the precipitation of protein from extracting solvents and enhance protein yield. This dual-stage ultrasound application will modify the protein structure, improve the quality, and functional characteristics of the protein. The novelty of this research lies in the dual-stage application of ultrasound in extraction and precipitation, which facilitates the extraction of rice bran protein with improved quality and functional properties. The primary objectives of this study are: (1) to evaluate the effect of ultrasound-assisted extraction (UAE) and ultrasound-assisted isoelectric precipitation (UIP) on the yield and purity of rice bran protein (RBP); and (2) to assess the effect of ultrasound on protein structure, functional, and nutritional properties of extracted protein. This dual-stage strategy is intended to overcome the limitations observed in single-stage ultrasound treatments and to enhance the overall functional quality and applicability of rice bran protein in food product development.

## 2 Materials and methods

### 2.1 Materials

The rice grain (medium grain cultivar XP5703) was obtained from the Arkansas Rice Processing Program, Fayetteville, AR, USA, collected during the harvest season, and stored at 4 °C. The bran was separated using a dehusker and polisher (FC2R-Y, Yamamoto, Japan). The rice bran was sieved to 250 µm and defatted using n-hexane in a 1:10 (w/v) ratio at room temperature for 2 h with continuous stirring. After defatting, it was centrifuged at 7000 × g for 25 min. The defatted residue was uniformly distributed for drying at ambient temperature with airflow for 8 h [11,12]. The nitrogen/ protein content of the rice bran was estimated using Lowry’s method, and fiber (acid- and neutral-digested) was calculated using the Weende method. The starch was estimated using the Megazyne starch assay kit (AA/AMG) and lipid content was analyzed using Soxhlet method.

### 2.2 Extraction of rice bran protein

#### 2.2.1 Alkaline extraction

Defatted rice bran was mixed with deionized water at 1:10 (w/v). The pH was carefully adjusted to 8.0, 8.5, 9.0, 9.5, and 10.0 using 1 M NaOH. The slurries were stirred continuously for 1 h and centrifuged (Beckman-Coulter J2 series, IN, USA) at 7000 rpm for 20 min. The supernatant was collected, and the protein concentration in the supernatant was determined using the Bradford method [20]. 10 µL of protein supernatant was mixed with 200 µL of Bradford dye (4 times diluted from Bradford dye concentrate, Bio-Rad Laboratories, IL, USA), rested for 5 min, and the absorbance was measured at 595 nm (BioteK H1M, CA, USA) [8]. Based on the results that pH 9.5 (Supplementary file 1, Fig. S3) exhibited the highest protein content, further experimentation was performed at an extraction pH of 9.5.

#### 2.2.2 Ultrasound-assisted alkaline extraction

**Fig. 1:**
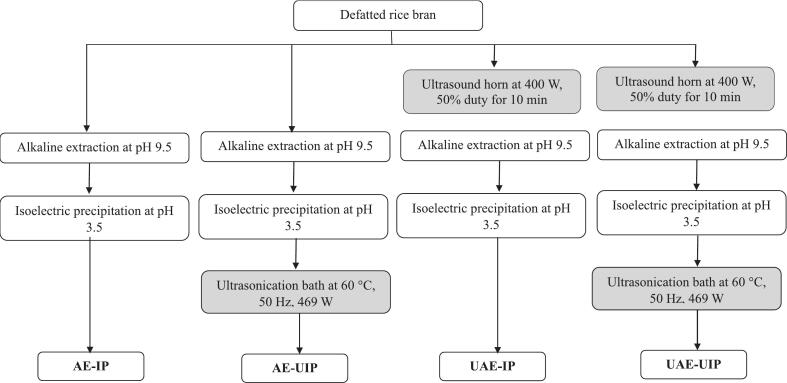
Schematic flow chart of RBP extraction. AE-IP: Alkaline extraction with isoelectric precipitation (Control); AE-UIP: alkaline extraction with ultrasound-assisted isoelectric precipitation; UAE-IP: ultrasound-assisted alkali extraction with isoelectric precipitation; and UAE-UIP: ultrasound-assisted alkali extraction with ultrasound-assisted isoelectric precipitation.

Ultrasound-assisted extraction of defatted rice bran (Fig. 1) was performed using a Branson 450 Series ultrasonic unit (Branson Ultrasonics Corporation, Danbury, CT), which has a 13 mm diameter of the horn tip with a maximum power output of 400 W. Ultrasound was applied from 1 to 10 min with a 50 % duty cycle. The optimized sonication conditions were 400 W, 50 % duty cycles for 5 min (Fig. S1) were used for this experiment.

The rice bran was suspended in deionized water at a 1:10 (w/v) ratio before sonication. An ice bath was used to control temperature between 20–25 °C (room temperature conditions) during sonication [14]. The pH of the sonicated mixture was then adjusted to 9.5, followed by stirring for 1 h. The mixture was centrifuged at 7000 rpm for 20 min at 4 °C to separate the supernatant, which was subsequently analyzed for protein content using the Bradford method (elaborated in previous sections).

#### 2.2.3 Isoelectric precipitation

To determine the optimal pH for protein precipitation, the supernatants obtained from alkaline extraction were acidified using 1 M HCl to pH levels ranging from 2.5 to 5.0. After precipitation, they were centrifuged at 7000 × g for 15 min. The supernatants were analyzed for their soluble protein content using the Bradford method [20]. The supernatants with the least protein were taken as the optimal pH for precipitation. The pH of 3.5 showed the least protein content in the supernatant (Fig. S2) and was used as the precipitation pH for the later experiments.

#### 2.2.4 Ultrasound-assisted isoelectric precipitation

After identifying the ideal precipitation conditions, ultrasound-assisted precipitation was performed at 60 °C, 50 Hz, 230 V, and 469 W for 1 h in an ultrasonic bath (Branson Ultrasonics Corporation, Danbury, CT) after adjusting the pH to 3.5. After sonication, the protein slurry was centrifuged at 7000 rpm for 20 min, and the pellet was collected afterwards. The extracted proteins were washed twice using distilled water adjusted to pH 3.5 and then freeze-dried. The dried residues were ground into a fine powder using a mortar and pestle and stored in airtight containers at 4 °C for further analysis. Fig. 1 explains the methods of extraction and precipitation in different combinations to elaborate on the experimental design of the current study.

#### 2.2.5 Protein recovery and yield (%)

The purity of the protein was evaluated using the Dumas combustion method [21]. The protein recovery % was calculated considering the mass yield of the dried protein isolates and purity %, as given below.

$$Protein\;\;recovery\;\;\left(\%\right)=\;\frac{Weight\;of\;the\;dried\;proteins\;\times Purity\;\%}{Total\;protein\;in\;defatted\;rice\;bran}\;\times 100$$

The purity of the protein was considered while making the solutions for all the functional and digestibility properties.

### 2.3 Structural changes in the proteins

#### 2.3.1 SDS-PAGE of extracted proteins

The extracted protein samples were subjected to gel electrophoresis using a method described by Rahman et al. (2020) with minor modifications. Protein samples were prepared at 1 mg/mL in sample buffer and heated at 100 °C for 10 min to denature the proteins. The cooled samples and a molecular weight marker (10–250 kDa) were loaded onto 4 % stacking/20 % separating gels. The electrophoresis was performed at 180 V for 60 min. The gels were stained for 40 min and then destained for 2 h. Finally, the gels were imaged and analyzed.

#### 2.3.2 Changes in secondary conformation using FT-IR

The secondary structural changes in proteins were analyzed using Fourier-transform infrared (FTIR) spectroscopy. An FTIR spectrometer (Cary 630, Agilent, USA) equipped with a diamond ATR accessory was used, operating at 4 cm^−1^ resolution at 4000–400 cm^−1^ [22]. A 1–2 mg sample was applied to the ATR crystal post-background acquisition. Second derivative spectra were analyzed using OriginPro 2017. The obtained spectra were processed by performing baseline correction, and all the secondary structures were individually isolated to derive the secondary spectra. The data were processed by fitting them to the Gaussian equation and obtaining the intensities of each secondary structure.

#### 2.3.3 Surface hydrophobicity

The surface hydrophobicity was conducted using the fluorescent method with 1-Anilinonaphthalene-8-sulfonic acid (ANS) [23]. Protein samples (0–1000 µg/mL) in 10 mM phosphate buffer (pH 7) were mixed with an 8 mmol/L ANS solution in darkness. Fluorescence intensity was measured (excitation 330 nm, emission 490 nm). The surface hydrophobicity index was determined from the slope of fluorescence intensity versus protein concentration.

### 2.4 Functional properties

#### 2.4.1 Protein solubility

The solubility profile of protein against various pH (2.0, 3.0, 4.0, 5.0, 7.0, 9.0, 10.0, and 12.0) was measured to evaluate the influence of pH on solubility [18]. 1 % (w/v) protein dispersions were prepared in distilled water, pH adjusted (2.0–12.0) and stirred for 1 h at room temperature. The 1 % (w/v) protein dispersions were prepared with consideration for protein purity to evaluate the functional properties. After centrifugation (7000 rpm, 15 min, 4 °C), supernatant protein concentration was determined by Bradford assay using Bovine serum albumin as a standard.

#### 2.4.2 Emulsifying properties

The emulsifying activity index (EAI) and stability index (ESI) of the protein emulsions was analyzed using a method described by Rahman et al. [18] with slight modifications. Emulsions were prepared by homogenizing corn oil with 1 % (w/v) protein purity-adjusted protein solution (1:3 ratio) at 10,000 rpm for 60 s. Diluted emulsion samples in 0.1 % SDS were measured spectrophotometrically at 500 nm at 0, 15, 30, 45, and 60 min. The EAI, expressed in m^2^/g, will be calculated as:

$$EAI=\;\frac{{2\times T}_{0}}{\varphi \times C\times 1000}$$

where $T_{0}$ is the initial emulsion turbidity, φ is the oil volume fraction (0.25), and C is the protein concentration in the dispersion (10 mg/mL). The ESI, expressed in minutes, will be calculated as:

$$ESI=\;\frac{A_{0}}{A_{0}-A_{t}}\times 100$$

where t is the time interval (15 min), and $A_{0}$ and $A_{t}\;$ is the absorbance values at 0 and 15 min, respectively.

#### 2.4.3 Foaming properties

To assess the foaming properties, the previously described method by Modupalli & Rahman [22] was adapted. 1 % (w/v) purity-adjusted protein solutions were prepared and vigorously agitated using homogenizer at 10,000 rpm for 1 min in graduated plastic tubes. The determination of foaming capacity (FC) was calculated by the volume difference before and after agitation, as following equation:

$$FC=\;\frac{Volume\;after\;agitation-Volume\;before\;agitation}{Volume\;after\;agitation}\;\times 100$$

The stability of the foam was evaluated using the same method, where the samples were allowed to sit for 15, 30, 45, and 60 min. The volume of foam was measured at each time intervals, and the foam stability (FS) was determined using:

$$FS=\;\frac{Volume\;of\;solution\;at\;time\;t-Initial\;volume}{Initial\;volume}\;\times 100$$

### 2.5 In-vitro protein digestibility

The in-vitro digestibility of RBP was assessed through a sequential-enzyme digestion method involving pepsin and pancreatin [24]. Gastric fluid was prepared by adjusting the pH of a 0.2 M sodium chloride solution to 2.0. Samples (2 mg/mL) were mixed with gastric fluid and incubated at 37 °C for 60 min, followed by the addition of pepsin (200 U/mg protein) (Sigma Aldrich, 3200U/mg) for another 60 min. After adjusting the pH to 7, pancreatin (0.4 × USP/mg protein) (Sigma Aldrich, 8USP/mg) was added, and the mixture was incubated at 37 °C for 120 min. Aliquots were collected at each digestion stage. Protein concentration was evaluated using the Bradford method as described in solubility section above, and digestibility was expressed as:

$$Digestibility\;\;\left(\%\right)=\left(1-\frac{Ph-Pb}{Ps}\;\right)\times 100$$

where Ph and Pb represent protein concentrations of the aliquots and blanks, respectively, and Ps represents the initial protein concentration.

The concentration of free amino acids was determined using a TNBS (2,4,6-trinitrobenzene sulfonic acid) assay [25]. A 50 μL aliquot of the digestate was mixed with 500 μL of 0.1 mM phosphate buffer and 500 μL of 0.05 % TNBS reagent, followed by incubation at 50 °C for 60 min. After cooling for 30 min, the absorbance was measured at 340 nm. Free amino acids were quantified against an L-Leucine standard curve and expressed as a percentage of the total amino acids in undigested samples.

### 2.6 Total amino acid analysis

Protein isolates (100 mg) were combined with 6 M HCl in a sealed container [26]. Samples were hydrolyzed at 110 °C for 24 h, neutralized with CaCO3, and derivatized. Amino acid analysis followed AOAC methods 989.30, 994.12, and 988.15. GC–MS analysis used an Agilent system with a Zebron ZB-AAA column. Injection was in split mode (40:1) at 280 °C. Oven temperature started at 110 °C for 1 min, then increased to 310 °C at 30 °C/min. Transfer line and ion source temperatures were 320 °C and 230 °C, respectively.

### 2.7 Amino acid scores and PDCAAS calculations

Protein Digestibility Corrected Amino Acid Scoring (PDCAAS) was calculated based on the WHO/FAO/UNU essential amino acid scoring pattern, using the protein digestibility values obtained via the in-vitro method for correction [27]. PDCAAS was calculated using Amino acid score (AAS) and protein digestibility.

PDCAAS=AAS×Proteindigestibility%

The AAS was calculated as given below:

$$\mathrm{AAS}=\frac{\mathrm{Limited}\;amino\;acid\;content\;in\;extracted\;proteins}{\mathrm{Same}\;amino\;acid\;content\;in\;refernce\;protein\;;soy}$$

In-vitro protein digestibility was used in calculations, and cysteine was taken as the limiting amino acid for RBPs using previous literature [28].

### 2.8 Statistical analysis

All the tests were performed as duplicates, and the results obtained were presented as mean ± standard deviation. The results were analyzed using ANOVA and Tukey’s test of significance (p < 0.05) with JMP 2.0 Statistical software (JMP Pro 17, JMP Statistical Discovery LLC, USA).

## 3 Results and discussion

The resulting protein isolates are categorized as AE-IP (alkaline extraction and isoelectric precipitation), AE-UIP (alkaline extraction and ultrasound-assisted isoelectric precipitation), UAE-IP (ultrasound-assisted extraction and isoelectric precipitation), and UAE-UIP (ultrasound-assisted extraction and ultrasound-assisted isoelectric precipitation).

### 3.1 Yield and purity of rice bran proteins

The proximate composition of the defatted rice bran was 40.84 ± 0.02 % starch, 11.65 ± 0.05 % protein, 31.00 ± 3.7 % acid-digested fiber, 8.03 ± 0.15 % neutral-digested fiber, and 1.24 ± 0.04 % lipid content.

**Fig. 2:**
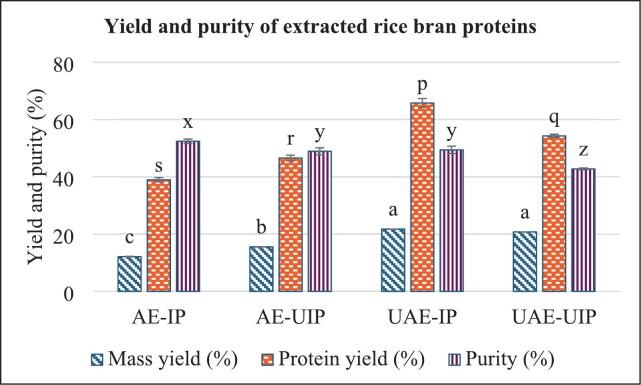
Protein recovery (%) of RBPs (Different lowercase letters within the same color column indicate a significant difference (p < 0.05). (AE-IP: Alkaline extraction with isoelectric precipitation (Control); AE-UIP: alkaline extraction with ultrasound-assisted isoelectric precipitation; UAE-IP: ultrasound-assisted alkali extraction with isoelectric precipitation; and UAE-UIP: ultrasound-assisted alkali extraction with ultrasound-assisted isoelectric precipitation).

The extraction and precipitation of rice bran proteins demonstrate that the mass yield and protein yield (extracted mass × purity% %) improved when ultrasound was applied in either the extraction or precipitation processes. The application of ultrasound impacts both protein yield and purity in rice bran protein extraction, as shown in Fig. 2a. Ultrasound-assisted extractions (UAE-IP and UAE-UIP) yielded significantly higher mass percentages of 22 % and 21 %, respectively, compared to the alkaline extractions (AE-IP and AE-UIP). This reiterates that ultrasound disrupts the rice bran particles, facilitating better protein extractability. However, the conventional method (AE-IP) achieved the highest protein purity of 52.40 %. The addition of ultrasound during extraction and precipitation reduced the purity of protein; the dual-stage, UAE-UIP exhibited the lowest purity of 41 %. This explains that the UIP stage facilitated the coagulation and precipitation of proteins and other dissolved materials from the supernatant. The protein yield showed that UAE-IP yields were 65.7 %, and there was no significant difference between AE-UIP and UAE-UIP. All samples treated with ultrasound (either UAE or UIP) have a higher overall protein yield than those obtained using the conventional method, primarily due to their higher mass yield. Therefore, ultrasound-assisted extraction disrupts the bran matrix to release proteins into the extraction liquid, while ultrasound-assisted precipitation precipitates proteins and other soluble substances from the extraction liquid. Consequently, it improves mass yield but reduces purity. Furthermore, the acid-digested fiber contents were found to be 18.9 ± 2.7 %, 18.7 ± 0.8 %, 8.8 ± 1.6 % and 1.8 ± 0.4 % for AE-IP, AE-UIP, UAE-IP, and UAE-UIP extracted rice bran proteins, respectively. This directly correlates with the protein purity and recovery of the extracted proteins.

This result is consistent with previous findings, which show a 22 % increase from 53 % in control alkaline extraction to 75.5 % in ultrasound-assisted alkaline extraction by Sun et al. [29]. Similarly, Ly et al. [17] reported that ultrasound extraction alone improved protein yields by 22.04 % compared to conventional methods. The enhanced yield can be attributed to cavitation, which disrupts protein-cell matrix interactions and facilitates the release of proteins. However, the application of ultrasound during precipitation helps form protein and protein-fiber complexes, rather than breaking them apart.

### 3.2 Structural changes in proteins

#### 3.2.1 Gel electrophoresis of extracted proteins

**Fig. 3:**
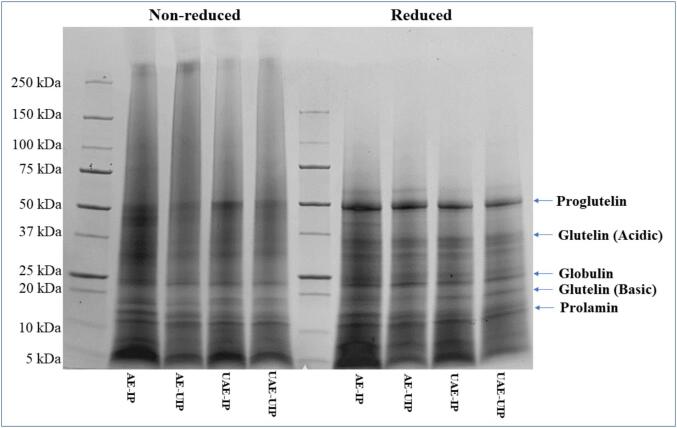
SDS-PAGE of extracted RBPs (AE-IP: Alkaline extraction with isoelectric precipitation; AE-UIP: alkaline extraction with ultrasound-assisted isoelectric precipitation; UAE-IP: ultrasound-assisted alkali extraction with isoelectric precipitation; and UAE-UIP: ultrasound-assisted alkali extraction with ultrasound-assisted isoelectric precipitation).

SDS-PAGE was performed to visualize the effect of ultrasound on the extraction and precipitation of proteins and their subunits (Fig. 3). The five major protein bands corresponding to prolamin (∼13–16 kDa), glutelin (22–23 and 37–39 kDa for basic and acidic subunits, respectively), globulin (∼26 kDa), and proglutelin (57 kDa) were observed (Fig. 3) [30]. Although the band patterns of the extraction methods were similar, the ultrasound treatment reduced the band intensity in reduced and non-reduced SDS-PAGE. Single-stage ultrasound, AE-UIP, and UAE-IP showed lower band intensities than the control AE-IP, while dual-stage UAE-UIP showed the least intense bands. These results may be attributed to the degradation of some amino acids or the disruption of hydrogen and other bonds during the ultrasound exposure [19]. Similar disruption of protein structure by the cavitation and bombardment effects during high-intensity ultrasound has been reported in previous studies [11,12,30,19].

#### 3.2.2 Secondary structure of proteins using FTIR

FTIR was used to understand the secondary structural change due to ultrasound treatment during the extraction and precipitation of proteins. Ultrasound triggers partial structural modifications that enhance protein functionality and yield [22]. The results showed that secondary structural components (amide II, β-sheets, random coils, α-helix, β-turns) decreased when ultrasound was used either during extraction or precipitation (Table 1 and Fig. S4). However, sonication treatment improved the β-sheets while reducing α-helices when applied for both extraction and precipitation. Similarly, it was found in literature that higher sonication power leads to more β-sheets and fewer α-helices in soy proteins [31].

**Table 1:** Ultrasound-assisted changes in the secondary structure of RBPs.

		Amide II	β-sheets	Random coils	α-helix	β-turns
AE-IP	Peak intensity	22.25 ± 0.75^a^	4.77 ± 0.20^b^	9.45 ± 0.25^a^	1.45 ± 0.23^a^	13.39 ± 0.42^a^
	% of total	43.37 %	9.30 %	18.42 %	2.83 %	26.08 %
AE-UIP	Peak intensity	8.31 ± 1.05^c^	2.77 ± 0.32^c^	5.32 ± 0.13^c^	1.51 ± 0.04^a^	14.04 ± 0.22^a^
	% of total	26.01 %	8.68 %	16.64 %	4.73 %	43.94 %
UAE-IP	Peak intensity	11.46 ± 0.51^b^	3.28 ± 0.22^c^	3.38 ± 0.27^d^	0.34 ± 0.08^c^	11.52 ± 0.45^b^
	% of total	38.21 %	10.94 %	11.29 %	1.14 %	38.42 %
UAE-UIP	Peak intensity	6.39 ± 0.51^c^	9.39 ± 0.28^a^	7.93 ± 0.44^b^	0.80 ± 0.06^b^	7.90 ± 0.40^c^
	% of total	19.72 %	28.98 %	24.46 %	2.47 %	24.37 %

Different lowercase letters within the same column indicate a significant difference (p < 0.05). (AE-IP: Alkaline extraction with isoelectric precipitation (Control); AE-UIP: alkaline extraction with ultrasound-assisted isoelectric precipitation; UAE-IP: ultrasound-assisted alkali extraction with isoelectric precipitation; and UAE-UIP: ultrasound-assisted alkali extraction with ultrasound-assisted isoelectric precipitation).

Ultrasound during extraction, UAE-IP significantly decreased amide II, β-sheets, random coils, α-helix, and β-turn components than the control, AE-IP. UAE-IP significantly decrease in β-sheets from 4.77 % to 3.28 % and a corresponding reduction in random coils from 9.45 % to 3.39 %. There were significant decreases also observed in α-helices from 1.45 % to 0.35 % and β-turns from 13.39 % to 11.53 % as compared to AE-IP.

However, ultrasound during precipitation, AE-UIP significantly decreases the β-sheets from 4.77 % to 2.77 % and random coils from 9.45 % to 5.32 % compared to AE-IP. There were significant differences in β-turns and α-helices between AE-UIP and control.

Applying ultrasound in both extraction and precipitation (UAE-UIP) significantly increased the β-sheet content from 4.77 % to 9.39 % due to the synergistic effects of ultrasound and precipitation. However, other structural elements such as random coils, α-helices, and β-turns exhibit significant decreases. The α-helices decreased from 1.45 % to 0.80 %, and β-turns from 13.39 % to 7.90 %. The β-sheet promoted protein reorganization into more ordered structures while nullifying the surface charge for aggregation and improving protein stability. The transition from α-helical to β-sheet structures involves the disruption of hydrogen bonds that stabilize α-helices [32]. It also allows the polypeptide chains to reorganize into β-sheets, generally exhibiting greater stability under sonication [18]. A similar transition from α-helices to β-sheets was observed when RBPs were extracted after ultrasonication, using alkaline extraction [32]. The application of ultrasound causes partial unfolding and transition of covalent interactions and thereby leads to secondary structural changes. This directly affects the protein's functional and nutritional properties, determining its product applicability [33]. Hence, it can be inferred that because of having one ultrasound step, either in extraction (UAE) or in isoelectric precipitation (UIP), the protein structure was loosened. However, the combined treatment weakened its rigidity while increasing its flexibility, which tended to decrease β-sheet content [34].

#### 3.2.3 Surface hydrophobicity

Surface hydrophobicity of proteins is quantitatively characterized by the exposure of hydrophobic amino acid residues on the protein's surface, which depends on protein denaturation or aggregation [31]. In this study, applying ultrasound to the extraction and precipitation alters surface hydrophobicity (Table 2). Ultrasound during extraction, UAE-IP demonstrated no significant change in hydrophobicity than the control. When ultrasound was applied during precipitation, both the AE-UIP and UAE-UIP methods showed a significant decrease in hydrophobicity, and dual-stage ultrasound during both extraction and precipitation (UAE-UIP) showed the least hydrophobicity.

**Table 2:** Surface hydrophobicity of RBPs.

Sample	Surface Hydrophobicity Index
AE-IP	56.22 ± 0.53^a^
AE-UIP	50.86 ± 0.53^b^
UAE-IP	57.38 ± 1.37^a^
UAE-UIP	36.63 ± 1.25^c^

Different lowercase letters within the same column indicate a significant difference (p < 0.05). (AE-IP: Alkaline extraction with isoelectric precipitation (Control); AE-UIP: alkaline extraction with ultrasound-assisted isoelectric precipitation; UAE-IP: ultrasound-assisted alkali extraction with isoelectric precipitation; and UAE-UIP: ultrasound-assisted alkali extraction with ultrasound-assisted isoelectric precipitation).

When ultrasound was applied during extraction in UAE-IP, the high-intensity, localized cavitation and shear forces, along with radical species, significantly disrupt the molecules. This intense mechanical agitation unfolds the proteins and exposes previously buried hydrophobic polar groups, increasing their surface hydrophobicity to 57.38. In addition, the application of subsequent ultrasound during precipitation UAE-UIP buried hydrophobic groups and decreased surface hydrophobicity to 36.63. Thus, the higher energy input from the ultrasound bath during precipitation fails to maintain the elevated hydrophobicity induced during the high-intensity extraction phase, resulting in a noticeable decrease in the overall hydrophobicity of the protein.

In summary, ultrasound disruption leads to exposure to buried hydrophobic groups, increasing surface hydrophobicity. Surface hydrophobicity tends to increase steadily with increasing ultrasound power up to a certain point as in UAE-IP [35]. However, excessive ultrasound treatment can lead to reaggregation of proteins, potentially decreasing surface hydrophobicity (UAE-UIP). Further correlation can be established based on the higher mass yield and lower purity of UAE-UIP. A study observed similar phenomena where excessive energy input leads to the aggregation of RBP protein molecules, which disrupts the exposure of hydrophobic groups [32]. This supports the notion that while moderate ultrasound treatment promotes the unfolding and exposure of hydrophobic groups, excessive power leads to protein aggregation and reduced hydrophobicity.

### 3.3 Functional properties

#### 3.3.1 Solubility

**Fig. 4:**
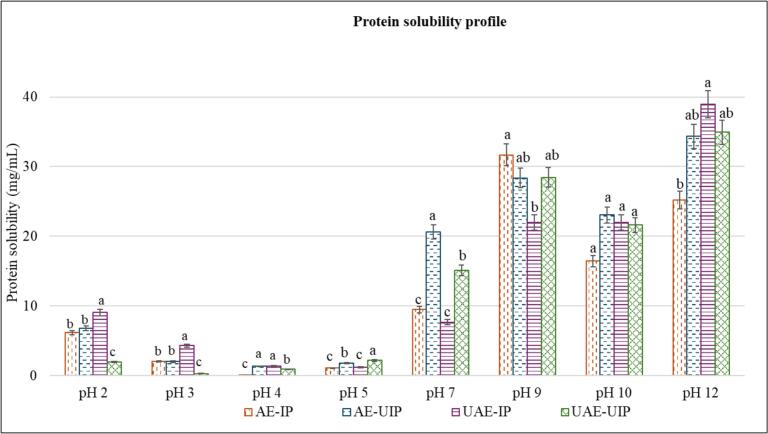
Solubility of RBPs as a function of pH (Different lowercase letters among the samples within the same pH indicate a significant difference (p < 0.05). (AE-IP: Alkaline extraction with isoelectric precipitation (Control); AE-UIP: alkaline extraction with ultrasound-assisted isoelectric precipitation; UAE-IP: ultrasound-assisted alkali extraction with isoelectric precipitation; and UAE-UIP: ultrasound-assisted alkali extraction with ultrasound-assisted isoelectric precipitation).

Conventional AE-IP’s solubility profile across pH 2–12 (Fig. 4) has not shown a traditional plant protein’s U or V-shaped solubility profile. It exhibited 5.3 mg/mL protein solubility at pH 2 and an isoelectric point of pH 3.5, whereas the highest solubility was 32.5 mg/mL at pH 9. Literature also showed similar isoelectric points of RBP (Fabian et al., 2010). The solubility of RBPs increased in both acidic and alkaline pHs. At acidic pH 2, UAE-IP showed the highest solubility of 9 mg/mL, while UAE-UIP showed the least, 2 mg/mL. Further, the solubilities of all samples were very low at pHs 3, 4, and 5 because of the isoelectric zone. UAE-IP exhibited significantly higher protein solubility at acidic pH (2 and 3) and basic pH of 12 than other samples. However, ultrasound-assisted precipitation (AE-UIP and UAE-UIP) showed significantly higher solubility at pH 7, ideal for most food product development. The highest solubility was 20.6 mg/mL by AE-UIP at pH 7, showing significantly lower surface hydrophobicity. Applying ultrasound at the alkaline pH (9–12) exhibited significant variability. At 9, AE-IP showed the highest solubility, while UAE-IP showed the least. Further, at pH 10, AE-UIP showed the highest, while AE-IP solubility was the least.

In summary, UAE-IP increased the solubility at acidic and basic pH, while AE-UIP exhibited the highest solubility at pH 7. Using ultrasound in both stages (UAE-UIP) also improved solubility, but not as much as the single-stage ultrasound applications. Interestingly, the UAE-UIP showed significantly higher solubility at pH 7.0 (almost doubled) compared to the control, which correlated with its lower surface hydrophobicity. According to López-Mártir et al. [36], the increase in protein solubility by sonication may be related to the decrease in hydrophobic groups on the protein surface. Furthermore, sonication promoted the formation of random coils, which are more conducive to solubilization. Applying ultrasound energy during extraction effectively disrupts intermolecular interactions, leading to the denaturation of structured proteins. This disruption facilitates the conversion of β-sheets, enhancing the exposure of hydrophilic regions, thereby increasing overall solubility [18].

#### 3.3.2 Emulsifying properties

The emulsification properties of plant proteins are crucial for stabilizing food products, such as mayonnaise and plant-based alternatives, thereby enhancing texture and shelf life. They provide a natural alternative to synthetic additives and animal proteins, supporting the growing demand for plant-based diets [37].

**Fig. 5:**
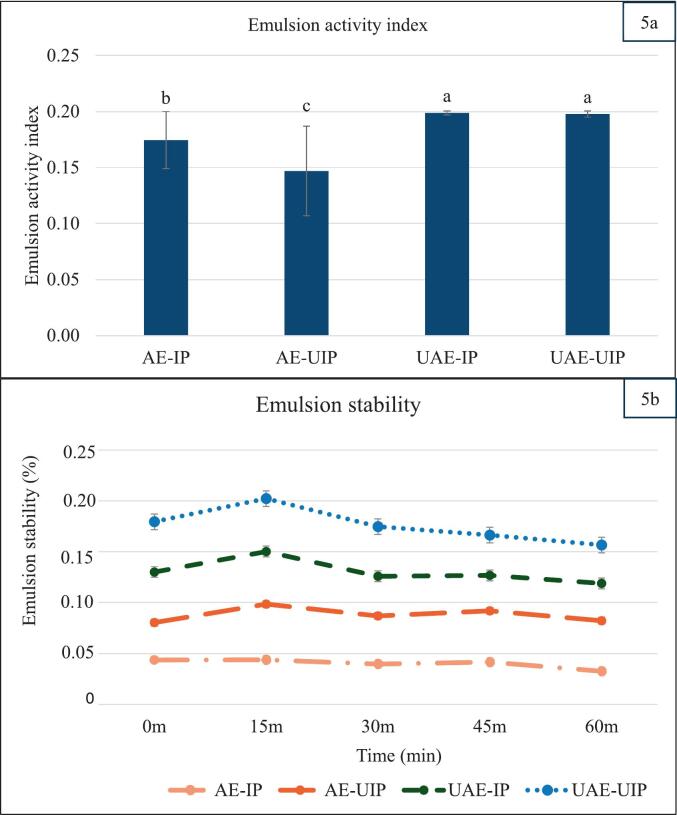
Emulsion properties of extracted RBPs (AE-IP: Alkaline extraction with isoelectric precipitation (Control); AE-UIP: alkaline extraction with ultrasound-assisted isoelectric precipitation; UAE-IP: ultrasound-assisted alkali extraction with isoelectric precipitation; and UAE-UIP: ultrasound-assisted alkali extraction with ultrasound-assisted isoelectric precipitation) (* The samples with different letters have a significant difference among each group, p < 0.05).

Emulsion activity: The results show that the application of ultrasound during extraction (UAE-IP and UAE-UIP) significantly increased the emulsion activity compared to alkaline extractions (AE-IP and AE-UIP) (Fig. 5(a)). There was no significant difference between UAE-IP and UAE-UIP; both exhibited an emulsion activity index (EAI) of 0.19. In contrast, AE-UIP exhibited the lowest emulsion activity of 0.15, compared to 0.17 of AE-IP. The addition of ultrasound during precipitation (AE-UIP) to alkaline extraction significantly decreased the emulsion activity, which can be correlated with the reduced hydrophobicity index. The improved hydrophobicity of UAE-IP increased the surface hydrophobic sites, which helped adhere to more lipid molecules. Further, more sulfhydryl groups are exposed to ultrasound, which can lead to improved interfacial network formation through disulfide bonds, thus improving emulsion formation [18]. The improved solubility also contributes to the increased emulsification after ultrasonic extraction [38]. Further, though the surface hydrophobicity of UAE-UIP drastically reduced, its higher solubility might have contributed to better emulsion formation.

Emulsion stability: Similarly, applying ultrasound during extraction and precipitation significantly increased the emulsion stability. UAE-UIP exhibited the highest emulsion stability, followed by single-stage ultrasound UAE-IP and AE-UIP. The emulsion stability of UAE-UIP was 0.2 after 15 min, which was significantly higher than other samples (Fig. 5(b)). The control (AE-IP) showed the least emulsion stability at 60 min. The addition of ultrasound-assisted (AE-UIP) led to an emulsion stability close to 0.1 after 30 to 60 min. UAE-IP showed a sharp and significant decrease after 15 min and was stable over 0.1 from 30 to 60 min. Similarly, dual-stage ultrasound extracted UAE-UIP showed a gradual decline throughout the incubation, with over 0.15 emulsion strength at the end of incubation. This reiterated better emulsion stability when ultrasound-assisted precipitation was involved. The decreased α-helices and random coils might be the reason for increased flexibility and exposure of binding sites. This increased exposure to hydrophobic groups, which improved solubility, was the primary reason for the improvement [39]. Ultrasound precipitation exposes more sulfhydryl groups to the aqueous solution, enhancing protein-fat interactions [29]. This is consistent with the findings of [40], who observed that ultrasound treatment significantly improved the water- and oil-holding capacities of plant protein. These enhancements were attributed to the formation of a spongy protein structure and increased exposure of hydrophobic regions, which improved interactions with surrounding components. While the enhanced emulsifying properties observed in ultrasound-treated samples are primarily linked to protein structural changes, the potential contributions of non-protein fractions should not be overlooked. Ultrasound-induced modifications can influence the interactions between proteins, fibers, and lipids, creating a more stable and uniform matrix. Notably, ultrasound has been shown to enhance protein–fiber interactions by promoting better dispersion, which helps keep emulsions stable [41]. Similarly, lipids can also strengthen protein structures, like in soy protein gels, making the emulsion more cohesive and less likely to separate [42]. Such effects may play a role in enhancing the emulsification behavior of the extracts in the current study, as fiber and lipid interactions significantly contribute to emulsion stabilization.

#### 3.3.3 Foaming properties

**Fig. 6:**
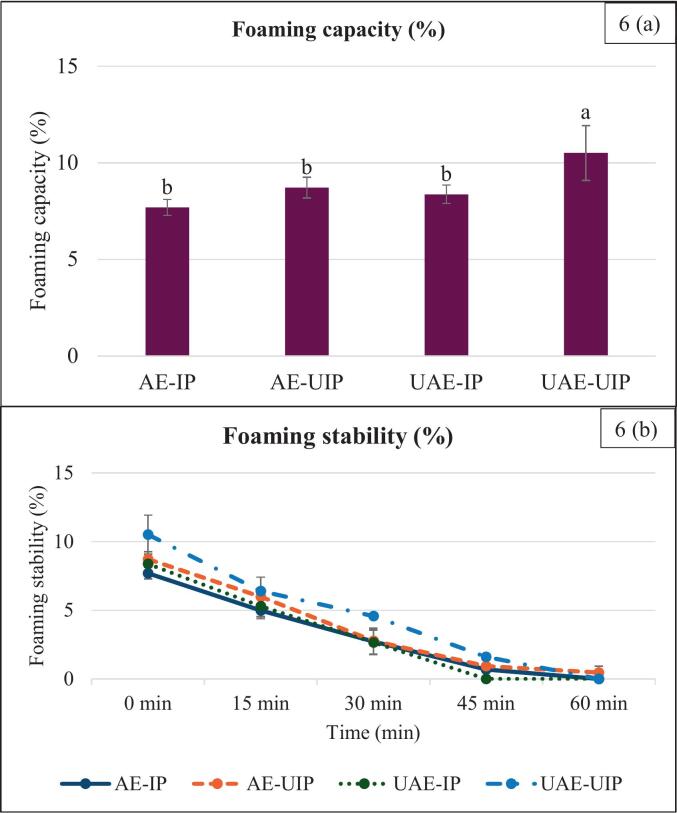
Foaming properties of extracted RBPs (Different lowercase letters within the same color column indicate a significant difference (p < 0.05). (AE-IP: Alkaline extraction with isoelectric precipitation (Control); AE-UIP: alkaline extraction with ultrasound-assisted isoelectric precipitation; UAE-IP: ultrasound-assisted alkali extraction with isoelectric precipitation; and UAE-UIP: ultrasound-assisted alkali extraction with ultrasound-assisted isoelectric precipitation) (* The samples with different letters have a significant difference among each group, p < 0.05).

The foaming stability and capacity of RBP extracted using different methods are closely related to its solubility. UAE-UIP exhibited better foaming properties, followed by single-stage ultrasound, i.e., UAE-IP and AE-UIP. UAE-UIP exhibited the highest foaming capacity of 10.51 %, whereas control AE-IP exhibited 7.5 % (Fig. 6(a)). However, there were no significant differences among the single-stage ultrasound samples, UAE-IP, and AE-UIP. This indicates that improved solubility enables better dispersion of proteins in the liquid, which is critical for effective foam formation. Furthermore, the decreased random coils and α-helices improved protein flexibility and interfacial properties.

Likewise, UAE-UIP exhibited the highest foaming stability after 60 min at 0.4 %, followed by AE-UIP and AE-IP at 0.9 % and 0.6 %, respectively, at 45 min. Foaming stability decreased significantly in the conventional UAE-IP method, dropping from 8.3 % at the start to 0 % after 30 min (Fig. 6(b)). In the case of UAE-IP, foam stability improved for the first 30 min and then decreased significantly compared to the control sample. The rapid decline in stability for AE-IP and UAE-IP can be attributed to decreases in solubility and protein dissociation, resulting in foam collapse.

While UAE-UIP exhibited the highest initial foam stability (10.51 %), AE-UIP ultimately retained the greatest stability (6.7 %) at the end of the 60-minute period, highlighting the beneficial role of ultrasound during precipitation. Dual-stage ultrasound, UAE-UIP breaks down clusters and creates flexible structures and improves solubility. This further enhances the surface hydrophilic sites, decreases the hydrophobicity index, and enhances foaming properties [43].

### 3.4 In-vitro digestibility

Ultrasonic cavitation can break down complex protein aggregates, exposing hidden peptide bonds to digestive enzymes. This increased surface area facilitates efficient protein hydrolysis, potentially improving amino acid absorption in the digestive tract [44]. In addition, cavitation also contributes to the disruption of cell walls in plant materials and makes them more accessible for digestion [45].

The digestibility of proteins indicated that ultrasound extraction and precipitation improved the gastric digestion of proteins (Table 3). The results showed that UAE-IP reduced digestibility by 10 %. However, ultrasound-assisted precipitation improved the digestibility by 17 % with AE-UIP and 31 % with UAE-UIP. A similar trend was observed during pepsin digestion, where UAE-IP showed a 7 % reduction. AE-UIP and UAE-UIP improved digestibility by 5 % and 4 %, respectively, compared to AE-IP. During pancreatin digestion, at the end of the first 60 min, there was a 4 % reduction in UAE-IP and a 6 % and 5 % increase in AE-UIP and UAE-UIP, respectively. However, in the second 60 min of pancreatin digestion, only AE-UIP showed an improvement in digestibility from 93 % of AE-IP to 96 %. The ultrasound-assisted extraction resulted in a 4 % reduction compared to conventional precipitation (UAE-IP) and a 21 % reduction with UAE-UIP. In summary, applying ultrasound during extraction and precipitation affected RBP digestibility differently. Ultrasound-assisted extraction by itself made the proteins harder to digest. On the other hand, ultrasound-assisted precipitation generally improved digestibility. However, one exception was when both techniques were combined in a dual-phase ultrasound (UAE-UIP); digestibility decreased during the second part of the pancreatin digestion phase.

**Table 3:** Digestibility and free amino acids during in-vitro digestion.

	**Gastric Fluid**		**Pepsin**		**Pancreatin-60 min**		**Pancreatin-120 min**	
	**Digestibility (%)**	**Free amino acids (%)**	**Digestibility (%)**	**Free amino acids (%)**	**Digestibility (%)**	**Free amino acids (%)**	**Digestibility (%)**	**Free amino acids (%)**
AE-IP	65.93 ± 2.11^c^	14.19 ± 0.00^a^	86.19 ± 0.80^b^	14.12 ± 0.16^a^	93.36 ± 0.34^b^	14.46 ± 0.26^a^	93.89 ± 0.63^b^	15.14 ± 0.32^a^
AE-UIP	83.66 ± 1.10^b^	14.50 ± 0.13^a^	91.97 ± 0.08^a^	14.53 ± 0.61^a^	96.10 ± 1.15^a^	15.05 ± 0.51^a^	96.91 ± 0.31^a^	15.07 ± 0.16^a^
UAE-IP	55.94 ± 1.41^d^	14.87 ± 0.26^a^	79.45 ± 1.20^c^	14.62 ± 0.03^a^	90.38 ± 0.42^c^	14.12 ± 0.03^a^	89.98 ± 0.42^c^	14.12 ± 0.10^c^
UAE-UIP	96.89 ± 0.07^a^	14.82 ± 0.20^a^	90.46 ± 0.64^a^	15.16 ± 0.42^a^	95.80 ± 0.04^a^	14.50 ± 0.13^a^	72.92 ± 0.18^d^	14.85 ± 0.03^b^

Different lowercase letters within the same column indicate a significant difference (p < 0.05). (AE-IP: Alkaline extraction with isoelectric precipitation (Control); AE-UIP: alkaline extraction with ultrasound-assisted isoelectric precipitation; UAE-IP: ultrasound-assisted alkali extraction with isoelectric precipitation; and UAE-UIP: ultrasound-assisted alkali extraction with ultrasound-assisted isoelectric precipitation).

While ultrasound extraction generally enhances the digestibility of plant proteins, certain factors can lead to reduced digestibility in some cases. Ultrasound-induced modifications to protein structure, such as alterations in secondary and tertiary structures, can sometimes negatively impact digestibility by making proteins less susceptible to enzymatic hydrolysis [46]. Additionally, the presence of other plant components, such as dietary fibers, may interact with proteins and form low-digestible complexes. Further, suboptimal ultrasound processing conditions, including excessive intensity or duration, can lead to protein degradation or the formation of indigestible compounds. The unchanged surface hydrophobicity makes it difficult for the enzyme to bind to the protein surface, thereby decreasing its reactivity [32]. These can be correlated with adverse changes in secondary structures. An increase in β-sheets and a decrease in α-helices created the orderliness of the structures, making it hard for the enzymes to bind at the active sites, reducing digestibility [44,35]. Moreover, the decrease in digestibility observed at 120 min could be attributed to the precipitation of hydrolyzed peptides during extended digestion periods. High molecular weight peptides and aggregates may precipitate over time, reducing their solubility and leading to an apparent decrease in digestibility as measured by standard assays [47,48].

Furthermore, by modifying protein structures and increasing enzyme-substrate interactions, ultrasonication can potentially increase the rate and extent of hydrolysis during digestion, along with the formation of free amino acids [49]. The study found that ultrasound treatments had varying effects on free amino acid content during digestion. During early digestion, including the gastric and pepsin phases and the first hour of pancreatin digestion, ultrasound slightly increased free amino acids without any significant difference. During the second hour of pancreatin digestion, AE-UIP produced results similar to the control method. However, using ultrasound-assisted extraction decreased free amino acids: a 0.98 % reduction when combined with conventional precipitation (UAE-IP) and a 0.29 % reduction when paired with ultrasound-assisted precipitation (UAE-UIP).

Ultrasound-induced structural changes might lead to the formation of peptide sequences that are more resistant to digestive enzymes, potentially reducing the release of some amino acids [44]. The changes in digestibility and free amino acids due to sonication can be correlated with the loss of secondary orientation of the proteins, along with the amino acid profile, in further sections.

### 3.5 Amino acid profile

RBPs offer a diverse amino acid profile, making them a valuable source of essential and non-essential amino acids. The protein fractions of rice bran contain significant amounts of glutamic acid, arginine, leucine, and valine, with a notable presence of branched-chain amino acids (BCAAs) [50]. The essential amino acid profiles vary among the protein samples, with AE-IP generally showing the highest levels for essential amino acids at 19.46 % (Table 4). The ultrasound precipitation with conventional extraction (AE-UIP) showed a significant decrease of 18.66 %. Further, ultrasound-assisted extraction with conventional precipitation showed no significant difference, while ultrasound-assisted precipitation significantly decreased from 19.46 % to 16.01 %. The UAE-UIP generally had the lowest levels of essential amino acids (compared to the other samples, which may impact overall protein quality. This can be correlated with the lowered band intensity in gel electrophoresis, which can be due to the cavitation effect causing breakdown of protein and amino acid structures. Despite variations, all sources contain all nine essential amino acids. This indicates that they are complete protein sources, though in different proportions. The data further shows varying levels of non-essential amino acids across the different protein samples (AE-IP, AE-UIP, UAE-IP, UAE-UIP), with glutamic acid consistently having the highest concentration among them, ranging from 5.89 to 7.06 %.

**Table 4:** Amino acid profile of RBPs.

**Amino acids**	**AE-IP**	**AE-UIP**	**UAE-IP**	**UAE-UIP**
Alanine	3.24 ± 0.01^a^	3.02 ± 0.10^a^	2.69 ± 0.45^a^	2.71 ± 0.03^a^
Arginine	5.05 ± 0.07^a^	4.84 ± 0.19^ab^	5.14 ± 0.01^a^	4.29 ± 0.09^b^
Aspartic acid	4.10 ± 0.04^a^	3.91 ± 0.13^ab^	4.13 ± 0.02 ^a^	3.50 ± 0.06^b^
Glutamic acid	6.95 ± 0.08^a^	6.70 ± 0.20^a^	7.07 ± 0.06^a^	5.89 ± 0.10^b^
Glycine	2.89 ± 0.05^a^	2.78 ± 0.09^ab^	2.95 ± 0.00^a^	2.47 ± 0.03^b^
Histidine	1.66 ± 0.03^a^	1.63 ± 0.03^a^	1.72 ± 0.01^a^	1.44 ± 0.02^b^
Isoleucine	1.88 ± 0.00^a^	1.78 ± 0.09^ab^	1.90 ± 0.01^a^	1.58 ± 0.03^b^
Leucine	3.83 ± 0.05^a^	3.66 ± 0.16^ab^	3.83 ± 0.04^a^	3.26 ± 0.05^b^
Lysine	2.88 ± 0.01^a^	2.73 ± 0.02^a^	2.88 ± 0.02^a^	2.39 ± 0.05^b^
Phenylalanine	2.39 ± 0.05^a^	2.36 ± 0.13^a^	2.45 ± 0.01^a^	2.04 ± 0.01^b^
Proline	2.06 ± 0.04^ab^	2.14 ± 0.06^ab^	2.26 ± 0.02^ab^	1.90 ± 0.04^b^
Serine	2.39 ± 0.04^ab^	2.31 ± 0.10^ab^	2.50 ± 0.05^a^	2.05 ± 0.03^b^
Taurine	0.01 ± 0.00^a^	0.01 ± 0.00^a^	0.01 ± 0.00^a^	0.03 ± 0.02^a^
Threonine	1.87 ± 0.04^a^	1.81 ± 0.09^a^	1.93 ± 0.05^a^	1.62 ± 0.04^a^
Tyrosine	1.68 ± 0.04^ab^	1.55 ± 0.06^ab^	1.73 ± 0.03^a^	1.45 ± 0.00^b^
Valine	2.96 ± 0.02^a^	2.89 ± 0.11^ab^	3.01 ± 0.01^a^	2.56 ± 0.04^b^
Cystine	0.76 ± 0.00^a^	0.71 ± 0.00 ^ab^	0.76 ± 0.02^a^	0.64 ± 0.02^b^
Methionine	1.05 ± 0.04^a^	0.92 ± 0.00^ab^	0.99 ± 0.01^a^	0.81 ± 0.01^b^
Tryptophan	0.94 ± 0.05^a^	0.88 ± 0.00^a^	0.71 ± 0.00^b^	0.60 ± 0.01^b^

Different lowercase letters within the same row indicate a significant difference (p < 0.05). (AE-IP: Alkaline extraction with isoelectric precipitation (Control); AE-UIP: alkaline extraction with ultrasound-assisted isoelectric precipitation; UAE-IP: ultrasound-assisted alkali extraction with isoelectric precipitation; and UAE-UIP: ultrasound-assisted alkali extraction with ultrasound-assisted isoelectric precipitation).

Lysine and threonine, the limiting amino acids in rice bran, showed different concentrations in different samples. The control sample contained 2.88 % lysine and 1.87 % threonine. The ultrasound extraction with conventional precipitation (AE-UIP) resulted in no significant changes in either amino acid. While ultrasound extraction with conventional precipitation (UAE-IP) had no effect on lysine content, it significantly increased threonine content. UAE-UIP reduced both amino acids significantly, as correlated with their essential amino acid contents. Leucine was the most abundant essential amino acid across all samples, showing no significant differences, with a range of 3.26 % to 3.83 %. Tryptophan consistently showed the lowest levels among all the essential amino acids. The data showed that ultrasound-assisted extraction reduced tryptophan content significantly (UAE-IP and UAE-UIP) compared to the control. However, there was no significant difference among alkaline extractions with different precipitations (AE-UIP and AE-IP). The data demonstrated that the choice of extraction method has a significant influence on the amino acid composition of the extracted protein isolates. The UAE-IP method showed promise in maintaining or slightly enhancing the amino acid content compared to the AE-IP. This could be attributed to the cavitation effect, which may have improved the breakdown of cell walls and enhanced protein solubilization. The data also suggests that UAE-IP maintains these levels well, which is essential for the nutritional value of the protein isolate. The high levels of glutamic acid and arginine, and relatively stable levels of cysteine and methionine across all methods, are also noteworthy. These amino acids are often limited in plant proteins, so maintaining their content is important for the overall protein quality and its ability to meet nutritional requirements [51].

During ultrasound treatment, protein deterioration, including R-group damage, is influenced by sono-chemical effects (like radical formation) and sono-mechanical effects (such as cavitation and shear stress), as well as factors such as ultrasound intensity, duration, temperature, and the protein's characteristics [52]. However, these trends appear method-specific, as the UAE-UIP combination showed the least favorable results.

### 3.6 Amino acid score and PDCAAS

**Fig. 7:**
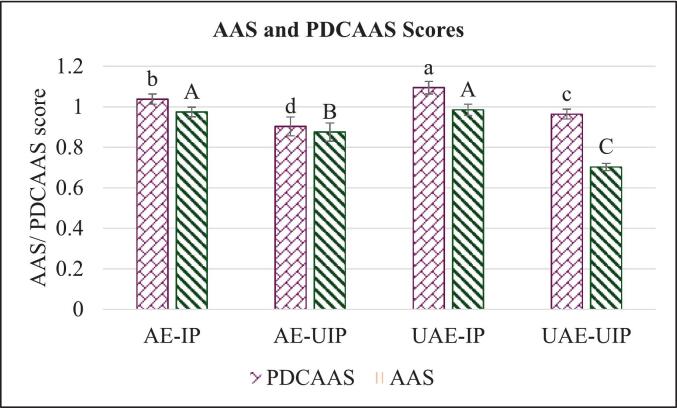
Amino acid score (AAS) and Protein-digestibility corrected amino acid score (PDCAAS) of extracted RBPs (Different lowercase letters within the same color column indicate a significant difference (p < 0.05). (AE-IP: Alkaline extraction with isoelectric precipitation (Control); AE-UIP: alkaline extraction with ultrasound-assisted isoelectric precipitation; UAE-IP: ultrasound-assisted alkali extraction with isoelectric precipitation; and UAE-UIP: ultrasound-assisted alkali extraction with ultrasound-assisted isoelectric precipitation) (* The samples with different letters have a significant difference among each group, p < 0.05).

PDCAAS (Protein Digestibility Corrected Amino Acid Score) is a crucial metric for evaluating the nutritional quality of plant proteins. The amino acid score measures the concentration of essential amino acids compared to human requirements. The results showed that PDCAAS showed no significant differences between the samples UAE-IP, AE-IP, and UAE-UIP (Fig. 7). However, AE-UIP had a significantly lower PDCAAS of 0.9 than other samples. Similarly, the AAS was similar for AE-IP, AE-UIP, and UAE-IP. Dual-stage ultrasound extracted UAE-UIP showed a significant decrease in AAS from 1.1 to 0.9, possibly due to excessive exposure to sonication. This can be correlated with the amino acid profile and essential amino acid content, as certain amino acids could have been oxidized during the sonication process. The PDCAAS values are comparable to those in previous literature, with a value of 0.81 for rice proteins [53]. The cavitation effects of ultrasound can partially unfold protein structures, which may improve digestibility by making the proteins more accessible to digestive enzymes [54]. Ultrasound extraction can be gentler than other conventional methods, potentially preserving more essential amino acids that contribute to a higher PDCAAS score [55]. In summary, the dual-stage application of ultrasound during RBP extraction significantly decreased the protein digestibility scores. In contrast, ultrasound during either extraction or precipitation did not affect AAS or PDCAAS.

### 3.7 Potential mechanism of protein extraction using ultrasound

**Fig. 8:**
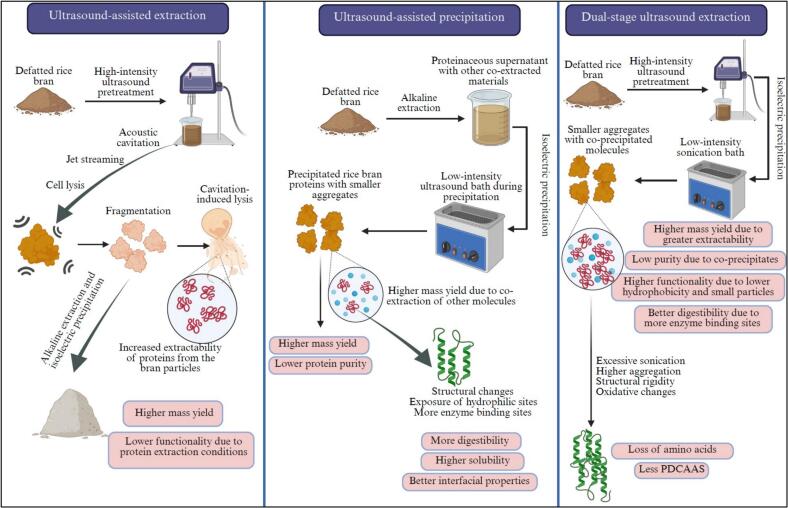
Proposed mechanism of dual-stage ultrasound application on protein yield and quality.

A proposed mechanism for the effects of ultrasound during the extraction and precipitation of rice bran proteins is described below and illustrated schematically in Fig. 8. When ultrasound is applied during the alkaline extraction stage (UAE), it significantly improves protein yield compared to the conventional methods. This improvement can be primarily attributed to the phenomena of ultrasonic cavitation and jet streaming that disrupt the cell walls of rice bran. This disruption facilitates the release of intracellular proteins into the extraction solvent at a higher rate. Simultaneously, acoustic streaming enhances solvent circulation and penetration into the bran matrix, further improving mass transfer from solid particles to the liquid phase. As a result, the UAE not only increases extraction efficiency but also induces notable changes in the secondary structure of rice bran proteins, which can enhance their solubility, emulsifying, and foaming properties.

When ultrasound is applied during the isoelectric precipitation stage, the acoustic cavitation and microstreaming effects generated by low-intensity ultrasound alter the aggregation and precipitation behavior of proteins. This process promotes the formation of protein–protein aggregates and increases the surface activity of protein, which can eventually trap or co-precipitate soluble non-protein constituents from the supernatant into the protein matrix. Although this co-precipitation slightly decreases the purity of the isolated proteins, it enhances the overall mass recovery. This process also disrupts secondary structural components and reduces surface hydrophobicity, exposing more hydrophilic groups on the protein surface. These structural modifications enhance the protein’s interaction with water and other molecules, thereby improving solubility, emulsification, and foaming properties.

The combined application of ultrasound during both extraction and precipitation stages (dual-stage ultrasound) offers synergistic benefits. Proteins obtained through this approach exhibit superior solubility, particularly at neutral pH, due to the formation of smaller aggregates, which promotes dispersion in water. Additionally, it contributes to improved emulsifying and foaming properties, making them more effective at interfaces. Ultrasound treatment also increases protein digestibility by exposing more sites for enzymatic hydrolysis, thereby facilitating the breakdown of proteins into their constituent amino acids. Despite these advantages, dual-stage ultrasound treatment can introduce some adverse effects. Prolonged exposure to ultrasonic energy may lead to excessive protein aggregation and increased structural rigidity, as evidenced by the changes in secondary structural components. Such changes can hinder enzyme access during digestion, potentially reducing protein digestibility and lowering the nutritional quality, as reflected by decreased amino acid scores. Therefore, while ultrasound-assisted extraction and precipitation can be highly effective for enhancing the yield and functionality of rice bran proteins, careful optimization of processing parameters is essential to maximize benefits while minimizing potential drawbacks.

## 4 Conclusion

In this study, ultrasound was applied at various stages of rice bran protein, which significantly affected the extractability and quality of the proteins. Ultrasonication at two different stages, extraction and precipitation, showed different impacts on the protein yield, molecular structure, and protein quality. While the conventional method showed higher protein purity, ultrasound-assisted samples show better mass yield, thereby, protein yield. The functional properties showed that protein solubility was improved by ultrasonic extraction at acidic and basic pHs and ultrasonic precipitation at basic pH. Emulsion activity and foaming stability improved with ultrasonic extraction, and emulsion stability and foaming activity increased with ultrasonic precipitation. The amino acid profile, however, showed a decrease in essential amino acid content, though all essential amino acids were present. The digestibility and PDCAAS scores improved with ultrasonic precipitations. The ultrasound-assisted extraction and precipitation method will increase the application of the rice processing byproduct in a wide variety of food products, such as plant-based alternate meat, plant-based dairy, whipped cream, and meringues that require good solubility, emulsion, and foaming properties. Their good amino acid profiles also help deliver complete protein in consumer-desired formulations. Despite the study's meaningful findings, it has several limitations, including that the energy consumption and processing costs of ultrasound treatment during extraction and precipitation were not measured, limiting the assessment of efficiency and scalability. The exclusive focus on rice bran protein differs from other plant proteins, such as soy, pea, and faba bean, which limits the generalizability of the results. Moreover, experiments were conducted only at laboratory scale, without addressing scale-up factors that may influence protein structure and functionality under industrial conditions.

## Data availability statement

The data that support the findings of this study are available from the corresponding author upon reasonable request.

## CRediT authorship contribution statement

**Saydul Md Safwa:** Writing – original draft, Software, Methodology, Investigation, Formal analysis, Conceptualization. **Nikitha Modupalli:** Writing – original draft, Software, Methodology, Investigation, Formal analysis, Data curation, Conceptualization. **Md Mahfuzur Rahman:** Writing – review & editing, Validation, Supervision, Resources, Project administration, Funding acquisition, Conceptualization.

## Declaration of competing interest

The authors declare that they have no known competing financial interests or personal relationships that could have appeared to influence the work reported in this paper.

## Data Availability

The data that support the findings of this study are available from the corresponding author upon reasonable request.
