# Supplementary material for: Dual-stage ultrasound application for rice bran protein extraction: A novel method to improve yield, functional properties, and nutritional profile
Source: Ultrason Sonochem. 2025 Nov 14;123:107681. doi: 10.1016/j.ultsonch.2025.107681 (PMC12666570; doi:10.1016/j.ultsonch.2025.107681)
Supplement: Supplementary Data 1 [file mmc1.docx]

**Supplementary data**

**Figure S1**: Protein concentration in the extraction liquid after different times of ultrasound exposure (* The samples showed no significant difference p<0.05)

**Figure S2**: Protein concentration in the supernatant after isoelectric precipitation at different pH levels (* The samples showed no significant difference p<0.05)

ab

ab

ab

b

a

**Figure S3**: Protein concentration in the supernatant after extraction at different pH levels (* The samples with different letters have a significant difference p<0.05)


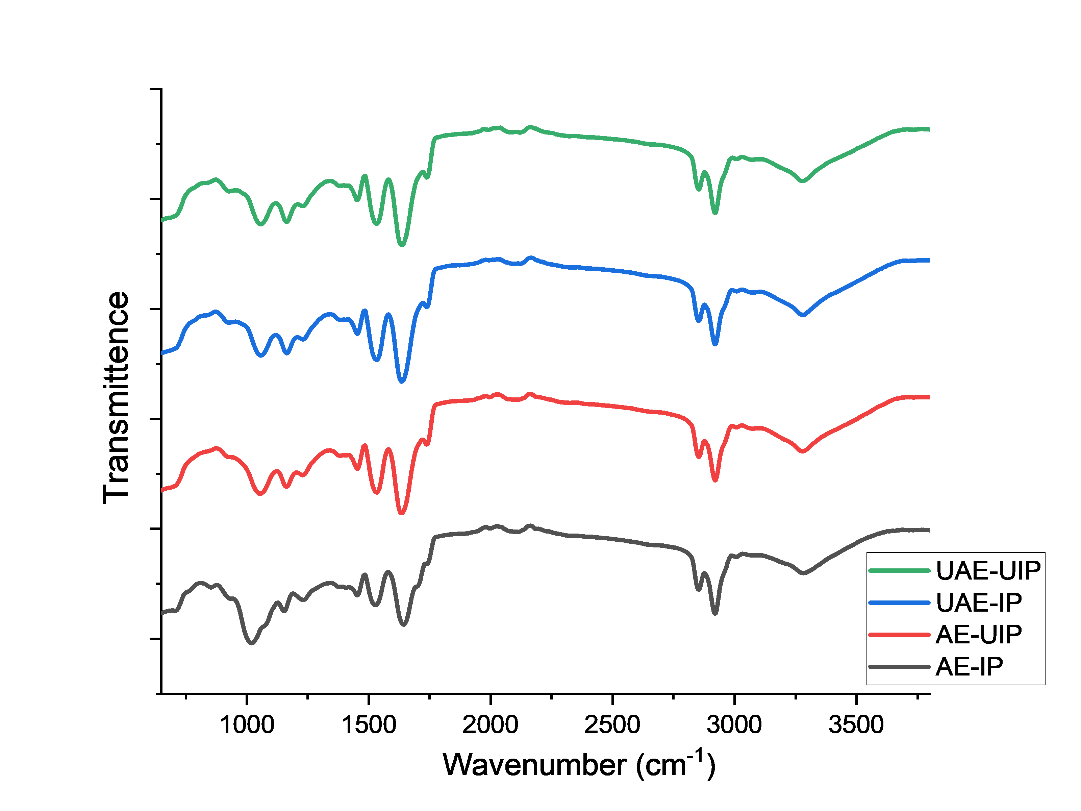


**Figure S4:** FTIR spectra of extracted proteins
